# Maternal, reproductive and obstetric factors associated with preterm births in Mulago Hospital, Kampala, Uganda: a case control study

**DOI:** 10.11604/pamj.2018.30.272.13531

**Published:** 2018-08-10

**Authors:** Elizabeth Ayebare, Peter Ntuyo, Oliver Ombeva Malande, Gorrette Nalwadda

**Affiliations:** 1 Department of Nursing, College of Health Sciences, Makerere University, Kampala, Uganda; 2 Department of Obstetrics and Gynaecology, Mulago Hospital, Kampala, Uganda; 3 Department of Paediatrics and Child Health, Egerton University, Egerton, Kenya

**Keywords:** Preterm birth, postpartum, risk factors, newborn, Uganda

## Abstract

**Introduction:**

Preterm birth, a leading cause of neonatal mortality accounts for 35 percent of all neonatal deaths worldwide. Uganda's high preterm birth rate of 13.6 per 1000 live births ranks 28^th^ in the world. Efforts at reducing these pre-term births must entail interventions that target any associated risk factors. This study therefore aimed at identifying and describing the risk factors for preterm births among mothers delivering in Mulago Hospital.

**Methods:**

This was a case control study among postpartum women in Mulago Hospital. Ninety nine women with preterm newborns were recruited as cases and 193 with full term babies were the controls. A semi-structured questionnaire was used to collect data. Data was entered into Epidata version 3.1 and exported to STATA 11 for univariate analysis and multivariate analysis by logistic regression.

**Results:**

Risk factors for preterm birth included maternal height less than 1.5 meters (OR 131.08 (20.35-844.02)), rural residence (OR 6.56(2.68-16.10)) and failure to attend antenatal care clinic (OR 8.88(1.44-54.67)). Pregnancy related risk factors included PPROM (OR 287.11(49.26-1673.28)), antepartum haemorrhage (OR 7.33(1.23-43.72)) and preeclampsia/eclampsia (OR 16.24(3.11-84.70)).

**Conclusion:**

Preterm birth is more likely to occur in women of short stature, living in rural areas and those who do not attend antenatal care clinic. The preterm birth risk is higher for women who get PPROM, APH and preeclampsia/eclampsia in pregnancy. Early recognition and management of these high risk conditions among pregnant women may lead to a reduction in preterm birth rates.

## Introduction

Preterm birth defined as delivery of a baby before 37 completed weeks of gestation, is associated with complications that lead to one million child deaths annually among neonates. It is also associated with increased risk of post neonatal mortality, long term neurological impairment, stunting and development of non-communicable diseases in adulthood [1]. Preterm birth is a major public health problem worldwide occurring in six to 10 percent of births in high income countries and up to 15 percent in low income countries. The highest burden (85%) for preterm birth is concentrated in Africa and Asia [2]. In sub-Saharan Africa, studies conducted in Gambia and Tanzania estimated the preterm birth incidence of 10.9 and 12 percent respectively [3, 4]. Uganda is the 28^th^ country worldwide with a high preterm births rate of 13.6 per 1000 live births [5]. These preterm births are directly responsible for 25 percent of the 27 neonatal deaths per 1,000 live births [6, 7]. In order to achieve the new global targets for neonatal mortality of less than 10 deaths per 1000 live births by 2035 [1], Uganda indeed has to worker harder than before to reduce preterm birth incidence. This will require identifying ways to address preventable causes of preterm birth as a priority in low income countries [2]. Whereas several demographic, social, obstetric and pregnancy related risk factors contributing to preterm births have been identified in other countries, these vary in different regions of the world with major disparities evident between high and low income countries [2]. However, there is limited evidence about the risk factors for preterm birth in the local setting that would be central in formulating preventive interventions. This study therefore examined factors associated with preterm birth so as to inform formulation of interventions for reducing preterm births.

## Methods

**Study design, site and population**: This was a case-control study conducted in Mulago Hospital postnatal ward and Special care Unit (SCU). Mulago hospital is the National referral and teaching hospital for Uganda located in Kampala city. The postnatal ward admits approximately 50-80 admissions daily of women who deliver from the labour ward. The SCU is the neonatal intensive care unit for the hospital. It admits preterm and sick full term babies born either in Mulago Hospital or in other neighbouring health centres. Women whose babies are in SCU remain on the postnatal ward until their babies are discharged. All women who gave birth either vaginally or by Caesarean section were recruited into the study. The New Ballard's Score (NBS) for estimating maturity was used to assess the gestational age of the newborns at birth [8]. Cases were selected using the LNMP based gestational age but at analysis, only those babies with NBS scores of 10 to 31 corresponding to gestational ages 28 to 37 weeks were considered as cases. The allocation to control or cases group was based only on the NBS score irrespective of their gestational age by LMNP.

**Case definition**: A preterm birth was defined as birth of a baby before 37 completed weeks of gestation [9]. All women who gave birth to preterm babies at hospital or at home, and were admitted in Mulago Hospital during the study period, within 72 hours after delivery and consented to the study were included. The controls were women who gave birth to full term babies from Mulago Hospital during the study period, within 72 hours after delivery and who consented to the study. Only women who were 72 hours postpartum or less were selected for the study to ensure accuracy of the NBS tool for estimating prematurity. We excluded all women who were very sick/weak and unable to answer the questions; those with critically ill babies.

**Sample size determination**: The Kelsey formula for estimating sample size for case control studies was used [10]. Basing on a study on risk factors associated with preterm births in Gaza, where attendance of less than 4 antenatal visits was a risk factor for preterm births [11] and a case to controls ratio of 1:2, we enrolled a total of 296 participants (99 cases and 197 controls). However, some of the data from controls was incomplete therefore only 193 participants were included in the analysis.

**Sampling procedure**: Consecutive sampling was used because of the limited number of preterm deliveries. Controls were selected following the cases from the birth register in the labour ward. Once a case was recruited, the two subsequent women with full term babies were selected as controls. In case a selected woman did not fit the criteria or declined to consent, the next woman on the register would be selected. Interviewer administered semi-structured questionnaires were used to collect information from the mothers enrolled in the study.

**Quality control**: A two days training for research assistants (qualified nurses) was conducted on topics such as; recruitment process, eligible participants, obtaining consent, filling the questionnaire, using the NBS, accessing information from the medical records and research ethics. The questionnaires were pre-tested among women who had delivered from a midwifery led labour ward and adjusted to capture the intended information.

**Data management**: Data was collected using a structured questionnaire, entered into a database using EPI DATA 3.1 and analysed using STATA version 11. Bivariate analysis was conducted on the socio-demographic, maternal and pregnancy related factors by comparing with the outcomes to obtain crude odds ratios. P-values below 0.05 were considered significant at confidence intervals of 95%. Maternal and infant characteristics were compared among cases and controls using descriptive statistics. Factors with a p-value of less than 0.1 were entered into logistic regression by backward stepwise method to identify factors independently associated with preterm birth.

**Ethical considerations**: This study was conducted in accordance with the declaration of Helsinki guidelines for protection of human participants. Approval to carry out this study was obtained from the institutional review board of the school of health sciences, Makerere University (SHSREC REF 2013-24) and Mulago Hospital Ethics Committee (MREC 400). Written informed consent was obtained from all the mothers enrolled in the study and serial numbers were used instead of participants' identifying information.

## Results

As presented in Table 1, the mean age of mothers with preterm births of 24 years (SD = 5.2) was similar to that of the controls: 24 years (SD = 5.3). The median maternal height was 1.56 (range, 1.2-2.0) metres for the cases lower than for controls which was 1.60 (range, 1.41-1.76) metres. As shown in Table 2, the mean gestational age according to the NBS was 32.7(SD = 2.4) weeks for the cases and 41.9(SD = 1.1) weeks for controls. The median age since birth for the babies was 22(range, 2-72) hours among the cases, higher than 14 (range 2-69) hours among the controls.

**Table 1:** Maternal socio-demographic characteristics

Variable	Case (n=99)	Control (n=193)	cOR(CI)	p-value
Mother’s Age*				
Less than 18yrs	4	9	0.86 (0.26- 2.85)	0.800
18yrs and above	95	183	1	
**Marital status**				
Married	88	173	1	
Single	11	20	1.08(0.51-2.36)	0.844
**Height (m)**				
Less than 1.5	35	3	**34.63(10.30-116.46)**	**<0.001**
1.5 and above	64	190	1	
**Pre-pregnancy BMI**				
Less than 25.0	53	147	1	
25.0 and above	46	46	**2.77(1.66-4.64)**	**<0.001**
**Post pregnancy BMI**				
Less than 25	47	126	1	
25.0 and above	52	67	**2.08(1.23-3.51)**	**0.003**
**Employment**				
Yes	60	92	1	
No	39	101	**0.59(0.36 - 0.97)**	**0.037**
**Residential area**				
Urban	40	143	1	
Rural	59	50	**4.22(2.44-7.29)**	**<0.001**
**Nature of Work**				
Light/Moderate Manual	83	172	1	
Heavy Manual	16	21	1.57 (0.73-3.36)	0.199
**Standing for >4 consecutive hours in a day**				
Yes	26	66	1.19(0.64-2.18)	0.549
No	42	127	1	
**Level of education**				
Primary and below	44	64	1.64(1.00- 2.70)	0.051
Secondary and tertiary	54	129	1	

* One missing age

**Table 2:** Demographic characteristics of newborns examined using New Ballard's score

Variables	Cases (%) n=99	Controls (%) n=193
Birth Weight*		
Low birth Weight <2500g	96(97.0)	9(4.7)
Normal birth weight ≥2500g	3(3.0)	183(95.3)
**Sex of baby**		
Male	42(42.4)	95(49.6)
Female	57(57.6)	98 (50.8)
**Mode of delivery**		
SVD	75(75.7)	92(47.7)
IVD	2(2.0)	63(32.6)
Elective C/S	6(6.1)	11(5.7)
Emergency	16(16.2)	27(14.0)
**Birth Type^α^**		
Singleton	81(81.8)	193(100)
Multiple	18(18.2)	0(0)

* Frequency is 291 due to 1 missing weight.

SVD- spontaneous vaginal delivery; IVD-Induced vaginal delivery; C/S-caesarean section

**Bivariate analysis**: Bivariate analysis results are presented in Table 1, Table 3 and Table 4. Factors found to be associated with preterm birth at bivariate analysis were maternal height less than 1.5 metres, body mass index equal or more than 25.0kg/m^2^, rural residence of mothers and failure to attend all the antenatal visits. Factors in the index pregnancy found statistically significant were; pre-eclampsia/eclampsia (p = 0.014), PPROM (p = <0.001), APH (p = <0.001), and trauma to the abdomen in index pregnancy (p = 0.033). No medical condition in pregnancy showed an association with preterm birth.

**Table 3:** Association between reproductive factors and preterm birth

Variable	Case (%)	Control (%)	cOR (CI)		P values
**Parity**					
Primipara	36(36.4)	83(43.0)	0.76(0.46-1.25)	0.275	0.275
Para 2- 9	63(63.6)	110(57.0)	1		
**Antenatal clinic attendance**					
Attended	91(91.9)	189(97.9)	1		
Did not attend	8(8.1)	4(2.1)	4.15(1.22 -14.15)	0.023	0.023
No. of antenatal visits*					
1- 3 visit(s)	72(79.2 )	83(43.9)	1		
4 or more visits	19(20.8)	106(56.1)	0.21(0.11 -0.38)	<0.001	<0.001
Gestational age at first ANC attendance**					
First trimester ≤12 weeks	20(22.0)	42(22.2)	1.01(0.55-1.84)	0.98	0.98
2^nd^ or 3^rd^ trimester	69(75.8)	146(77.8)	1		
Inter-pregnancy interval***					
≤12 months	6(9.2)	23(19.5)	0.42(0.16-1.09)	0.075	0.075
More than 12 months	59(90.8)	95(80.5)	1		
**Use of family planning methods**					
Yes	53(53.5)	75(38.9)	1.81(1.11-2.96)	0.017	0.017
No	46(46.5)	118(61.1)	1		

* Out of 280 participants who attended antenatal clinic.

** 2 participants not sure of age at first ANC attendance.

*** Frequency out of 186 participants

**Table 4:** Association between obstetric factors and preterm birth

Variable	Cases	Controls	cOR (CI)	p- value
Previous Pregnancy factors	n=66	n=120		
**Previous preterm birth**				
Yes	2	1	3.72(0.19-221.17)	0.287
No	64	119	1	
**Previous abortion**				
Yes	19	48	0.61(0.30-1.21)	0.128
No	47	72		1
**Bleeding before 28 weeks in previous pregnancy**				
Yes	1	4	0.45(0.01-4.64)	0.657
No	65	116	1	
**Previous still birth**				
Yes	4	7	1.04(0.21-4.29)	0.950
No	62	113	1	
**Index pregnancy obstetric factors**	**n=99**	**n=193**		
**PPROM**				
Yes	39	2	**62.08(15.08-537.86)**	**<0.001**
No	60	191	1	
**Antepartum Haemorrhage**				
Yes	12	4	**6.52(1.89-28.32)**	**<0.001**
No	87	189	1	
**Pre-eclampsia/eclampsia**				
Yes	9	5	**3.76(1.08-14.64)**	**0.014**
No	90	188	1	
**Bleeding before 28 weeks of gestation**				
Yes	8	11	1.45(0.49-4.12)	0.435
No	91	182	1	
**Incompetent cervix**				
Yes	4	3	2.67(0.44-18.50)	0.189
No	95	190	1	
**Trauma to abdomen**				
Yes	6	28	**0.38(0.12-0.98)**	**0.033**
No	93	165	1	

**Multivariate analysis of factors associated with preterm birth**: As presented in Table 5, Risk factors independently associated with preterm birth were height less than 1.5 metres (aOR = 131, 95% (CI: 20.35-844.02)); rural residence (aOR=6.56, 95% CI: (2.64-16.10)); being unemployed (aOR= 0.36, 95%(CI: 0.15-0.86)); failure to attend antenatal clinic (aOR=8.88 (95% CI: 1.44-54.67)); and PPROM, APH or preeclampsia/eclampsia in the index pregnancy with p-values of <0.001, 0.03 and 0.001 respectively .

**Table 5:** Crude and adjusted odds ratios of factors associated with preterm birth after logistic regression

Variable	Crude Odds Ratios (95%CI)	Adjusted Odds Ratio (95%CI)	P- value
**Height(metres)**			
Less than 1.5	34.63(10.30-116.46)	**131.08(20.35-844.02)**	**<0.001**
1.5 and above	1	1	
**Educational level**			
Primary or None	1.64(1.00-2.70)	1.46(0.61-3.49)	0.395
Secondary and above	1	1	
**Prepregnancy BMI (Kg/m^2^)**			
Less than 25.0	1	1	
25.0 and above	2.77(1.66-4.64)	1.49(0.62-3.60)	0.372
**Employment status**			
Employed	1	1	
Unemployed	0.59(0.36-0.97)	**0.36(0.15-0.86)**	**0.021**
**Residential area**			
Urban	1	1	
Rural	4.22(2.44-7.29)	**6.56(2.68-16.10)**	**<0.001**
**Antenatal attendance**			
Attended	1	1	
Did not attend	4.15(1.22-14.15)	**8.88(1.44-54.67)**	**0.019**
**Family planning Use**			
Yes	1.81(1.11-2.96)	2.11(0.86-5.20)	0.104
No	1	1	
**PPROM**			
Yes	62.08(15.08-537.86)	**287.11(49.26-1673.28)**	**<0.001**
No	1	1	
**APH**			
Yes	6.52(1.89-28.32)	**7.33(1.23-43.72)**	**0.029**
No	1	1	
**Preeclampsia/Eclampsia**			
Yes	3.76(1.08-14.64)	**16.24(3.11-84.70)**	**0.001**
No	1	1	
**Trauma to the abdomen**			
Yes	0.38(0.12-0.98)	0.20(0.36-1.05)	0.057
No	1	1	

## Discussion

The mean age of mothers of preterm newborns and that of full term newborns was comparable at 24 years. This could be due to the fact that the median age at first birth in Uganda is 19 years and the study included both primiparous and multiparous women [6]. Women with height less than 1.5 metres were more likely to have preterm birth as opposed to their taller counterparts. Although most studies [3, 12] have shown a positive association between maternal height and small for gestational age babies, an association with preterm birth also exists. In Norway and Gaza women of short stature (less than 1.63metres) had a higher likelihood for getting a preterm birth than taller women [11, 13]. Short stature may be a reflection of previous poor socioeconomic conditions and inadequate nutrition during childhood and adolescence [6]. In this study being unemployed reduced the chances of getting a preterm birth by 64 percent. On the contrary, a case control study in Portuguese maternities found that women getting pregnant while unemployed were 1.5 times more likely to have preterm births than working women [14]. Type of employment may however have an impact on a pregnant woman depending on whether it is manual or labour intensive. In Korea, women employed in jobs with manual labour were more likely to have preterm births compared to those in non-manual work [15]. In 2011, the UDHS showed that only 69 percent of Ugandan women were employed. In addition, majority (57%) of women were reported to work in the agricultural sector which is labour intensive while only five percent were in managerial positions [6]. Women residing in rural areas were more likely to have preterm birth in this study. Other studies have also shown that women residing in areas of lower education levels, with manual work and far from health facilities like rural areas are more likely to have poor birth outcomes [16, 17]. This is contrary to a study in Beinjing which found women in cities and urban centres to be more likely to have preterm births [18]. In Uganda, rural women are more likely to be of low education, involved in manual work such as farming and have poor access to health facilities.

Studies in Beinjing, Gaza and USA established the increased risk of preterm birth among women who do not get antenatal care in line with findings of this study [12, 18, 19]. In Zimbabwe also, lack of antenatal care attendance was found to double the risk of preterm birth [20]. Interventions during antenatal care such as CenteringPregnancy and other health education interventions were shown to reduce preterm birth rates by 47 percent in a low income setting [21]. Preterm premature rupture of membranes was independently associated with preterm birth. Moreover, it has been associated with 40 to 45% of all preterm deliveries [22]. This could be due to the reduced ability to prolong pregnancies due to facility and individual factors in low income countries. Similar to studies conducted in Gambia and Zimbabwe, women who had experienced antepartum haemorrhage were more likely to have a preterm birth in this study [4, 20]. Although APH is one of the danger signs of pregnancy which are communicated to pregnant women during antenatal care, its management depends on how quickly the woman gets to a health facility. In the case of placenta previa, it would be diagnosed on ultrasound in the third trimester but this is not common practice in Uganda. Pre-eclampsia/eclampsia is a major cause of poor birth outcomes worldwide including preterm birth, intra uterine growth retardation and intra uterine foetal death. Studies have shown preeclampsia/eclampsia to be another cause of medically induced preterm delivery [23]. This study found that mothers with preeclampsia or eclampsia were 16 times more likely to have a preterm birth. Studies in Gambia and Beinjing and Australia found similar findings [4, 18, 24].

**Limitations**: This being a case control study in design, recall bias could have occurred where some of the mothers were unable to remember some events in their previous pregnancies or even in the index pregnancy. Self-report was used for most of the parameters such as malaria during pregnancy which could have affected the findings. This limitation was overcome by review of the participants' medical records. Including primiparous women in this study may have reduced the ability of this study to establish relationships between previous pregnancy factors and preterm birth.

## Conclusion

Women with height < 1.5 metres; those residing in the rural areas; who did not attend antenatal care are more likely to have preterm births. The same is true for women who get preterm premature rupture of membranes, antepartum haemorrhage and preeclampsia. Early identification and prompt management of these factors can help reduce the rates of preterm births.

### What is known about this topic

- Preterm birth is one of the leading causes of neonatal mortality;
- Some of the intrapartum risk factors include preeclampsia, preterm premature rupture of membranes and antepartum haemorrhage;
- Interventions to prevent preterm births have been suggested including use of antenatal corticosteroids and tocolytics in preterm labour.

### What this study adds

- First, maternal height is a risk factor for preterm birth in the Ugandan setting;
- Secondly, antenatal attendance is still a key factor in reducing adverse pregnancy outcomes such as preterm birth;
- Lastly, multiple pregnancies present a real risk for preterm birth therefore, they should be managed in health facilities with adequate facilities for care of preterm babies.
